# Therapeutic Effects of Modified Si-Miao-Yong-An Decoction in the Treatment of Rat Myocardial Ischemia/Reperfusion Injury

**DOI:** 10.1155/2022/1442405

**Published:** 2022-06-06

**Authors:** Chen Wang, Yahong Wang, Dandan Song, Jing Su, Fangyuan Zhang

**Affiliations:** ^1^Institute of Basic Theory of Traditional Chinese Medicine, China Academy of Chinese Medical Sciences, Beijing, China; ^2^Dongzhimen Hospital, Beijing University of Traditional Chinese Medicine, Beijing, China; ^3^Department of Pathophysiology, Chinese PLA General Hospital, Beijing, China

## Abstract

**Objective:**

Modified Si-Miao-Yong-An decoction (MSMYA) was empirically originated from Si-Miao-Yong-An Decoction, which has been utilized for centuries to treat vasculopathy as well as heart diseases through clearing heat and detoxifying. This study aimed at confirming MSMYA's therapeutic effects for treating myocardial ischemia/reperfusion (I/R) injury and its underlying mechanisms.

**Methods:**

Rats were intragastrically administered with MSMYA for 4 weeks after ischemia/reperfusion (I/R) operation. Superoxide dismutase (SOD) and malondialdehyde (MDA) concentration were determined by calorimetry. Coagulation function was determined using an automated coagulation analyzer. Levels of cysteinyl aspartate specific proteinase (caspase)-1, interleukin (IL)-1*β*, interleukin (IL)-18, and lactate dehydrogenase (LDH) were measured by an enzyme-linked immunosorbent assay (ELISA). Infarct size was determined by triphenyltetrazolium chloride (TTC) staining. Myocardial histopathological and ultrastructure changes were examined by H&E staining and electron microscopy, respectively. Relative mRNA expression of NLRP3, an apoptosis-associated speck-like proteins containing the caspase activation and recruitment domain (ASC), caspase-1, IL-1*β*, and IL-18 were analyzed using quantitative real-time polymerase chain reaction (PCR). Meanwhile, their relative protein expressions were measured using western blotting.

**Results:**

The results showed MSMYA can inhibit oxidative stress by increasing SOD and reducing MDA, suppress inflammatory reaction by decreasing NLRP3 inflammasome-related cytokines' level, improve coagulation function by increasing prothrombin time (PT) and activating partial thromboplastin time (APTT), and ameliorate myocardial histopathological and ultrastructural changes. In addition, MSMYA's cardioprotective effects probably related to suppressing NLRP3 inflammasome pathway activation by reducing NLRP3 inflammasome molecular mRNA and protein relative expression.

**Conclusion:**

The results indicated that MSMYA played an important role in protecting the myocardium from I/R injury. The likely mechanism is the inhibition of oxidative stress, improvement of cardiac injury, and the reduction of NLRP3-related inflammatory cytokines release. This provides a basis for further research on the mechanism and clinical application of MSMYA to improve myocardial I/R injury.

## 1. Introduction

Acute myocardial infarct (AMI) remains the principal cause of hospitalization, disability, or death of coronary heart disease (CHD) worldwide. Percutaneous coronary intervention (PCI) is still an effective treatment for AMI [1]. However, studies have demonstrated that although reperfusion treatment releases symptoms and minimizes the area of infarction, the fatality rate does not apparently decrease and also provokes a series of events that lead to damage to myocardial function, metabolism, and electrophysiology, which is known as myocardial ischemia/reperfusion (MI/R) injury [2].

Myocardial I/R injury triggers several endogenous “danger signals,” such as oxidative stress, lysosome destabilization, and mitochondrial dysfunction that release damage-associated molecular patterns (DAMPs), which will trigger and recruit abundance of inflammatory cells, in turn, aggravates the heart injury. In recent years, many research studies reveal that inflammation is the key response although the whole process of CHD [3]. Myocardial injury can be exacerbated by an intense and highly specific inflammatory response, which occurs during reperfusion [4]. The NLR family pyrin domain-containing 3 (NLRP3) inflammasome, which is comprising NLRP3, an apoptosis-associated speck-like protein (ASC) and cysteinyl aspartate specific proteinase (caspase) 1, is the most widely studied cytosolic protein complex in inflammatory response, and its role in inflammation response is highlighted. Notably, the NLRP3 inflammasome is assembled in response to DAMPs. Recently, it has been reported that activation of the NLRP3 inflammasome is associated with the pathogenesis of ischemia/reperfusion (I/R) process besides numerous inflammatory diseases, and it may regulate the secretion of several proinflammatory cytokines, such as IL-1*β* and IL-18. Furthermore, inhibition of the NLRP3 signaling pathway has been shown to protect against myocardial ischemic injury [5].

The myocardial protective effect of Chinese medicine has been convinced, but the precise mechanisms are still unclear. Our main purpose is to investigate the therapeutic effects of modified Si-Miao-Yong-An (MSMYA) decoction, which is based on the original prescription but adding supplement qi and nourishing yin herb “American ginseng.” The effects of Si-Miao-Yong-An decoction on coronary heart disease (CHD) are certain and widely studied, especially in anti-inflammation [6–9]. Recent study has revealed the cardiomyocyte protective effects of Si-Miao-Yong-An decoction through activating autophagy and inhibiting NLRP3 inflammation-related pyroptosis [10]. Our previous studies also proved effects of Si-Miao-Yong-An on preventing myocardial ischemia injury by improving blood coagulation function and vascular endothelial function, reducing inflammatory cytokines release, cardiomyocytes apoptosis, and autophagy-related protein, as well as regulating apoptosis-related protein expression. Reperfusion injury is the main reason to restrict therapeutic effects of ischemia treatment, and in the clinic, we found that patients after PCI had apparent symptoms of qi or yin deficiency. “Deficiency” is the root pathogenesis of chest obstruction; therefore, supplement (qi or yin) deficiency should be strengthened as well as heat and detoxification should be cleared. American ginseng is the representative herb of supplement qi and yin. The modern pharmacological studies also showed that its active component Panax quinquefolius saponins had myocardial protective effects of improving hemorheology and blood circulation after ischemia/reperfusion injury [11, 12]. Our initial clinical study showed that MSMYA released chest pain of patients who suffered unstable angina pectoris. However, we did not clearly know its therapeutic effect on reperfusion injury. Based on that, we used MSMYA to investigate its effects on the rat myocardial ischemia/reperfusion injury model through different aspects such as anti-inflammation, antioxidative stress, improved coagulation function, histomorphology, and molecular biology.

In this study, we performed the in vivo I/R injury rat model to prove the protective effects of MSMYA on myocardial injuries. The main objective of this study is to investigate the pharmacological effects of MSMYA on MI/R and provide the experimental basis for clinical application.

## 2. Materials and Methods

### 2.1. Materials and Reagents

Modified Si-Miao-Yong-An Decoction (MSMYA) is composed of five components, as shown in Table 1. All herbs were purchased from China Beijing Tongrentang Group Co., Ltd. (Tongrentang, Beijing, China) and prepared in accordance with the Chinese pharmacopeia method (Chinese Pharmacopoeia Commission, 2015). The raw components were smashed and mixed with a weight ratio of 5 : 4 : 4 : 3 : 5. The material mixture was extracted twice with 8 times of water (vol/wt) by refluxing for 2.0 h each time. According to the clinical conversion method recorded in the book of “pharmacological methodology,” the drug concentration was determined by the clinical application dose, 70 kg adult taking 89 g/day crude herb, and the coefficient is 0.018 (for 200 g rats). Therefore, the dose for rats is 89 g x 0.018/0.2 kg = 8010 mg/kg/d = 8.01 g/kg/d. We applied 8 g/kg/d for the low dose and 1.6 g/kg/d for the high dose. The liquids were mixed and evaporated to a final concentration of 0.8 g/ml, which equals 1 ml of the liquid containing an equivalent of 0.8 g dried crude herbs. Extractions were filtered and stored at 4°C until used. Caspase-1, IL-1*β*, and IL-18 ELISA kits (Nanjing Jiancheng Bioengineering Institute, Nanjing, China), triphenyltetrazolium chloride (TTC) (Sigma, #T8877), SYBR Green qPCR Master Mix (MCE, #HY-K0501), anti-IL-1*β* antibody (Abcam, #ab9722) and anticaspase-1 antibody (Abcam, #ab286125), anti-NLRP3 antibody (NOVUS,#NBP2-12446), anti-ASC antibody (Santa Cruz, #sc-514414), anti-*β*-actin antibody (CST, #4967), enhanced chemiluminescence kit (Millipore, #00112878), HRP-conjugated bovine anti-mouse IgG (Santa Cruz, #sc-2375), and mouse anti-rabbit IgG (Santa Cruz, #sc-2357).

### 2.2. Animals and Treatment

Male adult Sprague Dawley rats (mean body weight mass 150 ± 20 g) were purchased from Beijing Vital River Laboratory Animal Technology Co., Ltd. (certificate no: SCXK (Beijing) 2016-0006, Beijing, China). Rats were raised in the animal experiment center at the Academy of Military Medical Sciences at temperature of 23°C ± 2°C and a relative humidity of 55% ± 10% under a 12 h/12 h light-dark cycle; they were given free access to water and a standard laboratory diet. All procedures in this study were approved by the Experimental Animal Ethics Committee of National Beijing Center for Drug Safety Evaluation and Research (approval no: IACUC-2020-007W) and were performed in accordance with the Guide for the Care and Use of Laboratory Animals published by the National Institutes of Health (NIH Publications, no. 8023, revised 1978).

Modified I/R protocol is as follows [13]: the rats were fixed on the operating table under anesthetization condition with 2% pentobarbital sodium peritoneally (2.3 ml/kg). Under artificial ventilation with a rodent ventilator, the thoracic cavity was opened at the third or fourth left intercostal space to expose the heart. The proximal portion of the left coronary artery (LAD) was surgically occluded for 45 minutes through ligation with a suture (size 6.0) followed by coronary reperfusion through release of the tie. Coronary occlusion was confirmed by elevation of the ST segment on the ECG obtained from a limb lead. The rats were returned to their cages.

The animals were randomly divided into six groups: the control group (*n* = 15), sham group (*n* = 15), model group (I/R, *n* = 15), NLRP3 inhibitor group (MCC950, *n* = 15), high-dose MSMYA group (HMSMYA, *n* = 15), and low-dose MSMYA group (LMSMYA, *n* = 15). The rats in the sham group underwent the same surgical procedure except for the ligation of the left coronary artery; according to reference [14], the rats in the MCC950 group underwent the same surgical procedure as the I/R group except for i.p adminstration 10 mg/kg MCC950 (NLRP3 inhibitor) solution every other day after operation. The rats in the MSMYA-treated groups were administrated with 0.8 g/kg/ml (low dose) and 1.6 g/kg/ml (high dose), respectively, for 4 weeks after I/R operation. The rats in the control group allowed free access to water and food until the end of the experiment. All treatments were given via gavage daily.

### 2.3. Sample Collection and Analysis

#### 2.3.1. Determination of Serum Oxidative Stress Cytokines

After the experiment, blood was taken from the abdominal aorta. The serum was separated by centrifugation at 3,000 rpm/min (4°C) for 15 min, and superoxide dismutase (SOD) and malondialdehyde (MDA) concentrations were determined by calorimetry.

#### 2.3.2. Determination of Serum and Myocardium Inflammatory Cytokines

After the experiment, blood was taken from the abdominal aorta. The serum was separated by centrifugation at 3,000 rpm/min (4°C) for 15 min, and heart tissue homogenate was prepared by homogenization of 10 mg tissue in 100 *μ*l of saline solution at 4°C. The serum inflammatory and myocardium tissue homogenate inflammation cytokines of cysteinyl aspartate specific proteinase (caspase) 1, IL (interleukin)-1*β*, IL-18, and lactate dehydrogenase (LDH) concentrations were measured using ELISA kits.

#### 2.3.3. Determination of Plasma Coagulation Function

After the experiment, blood was taken from the abdominal aorta. The plasma was separated by centrifugation at 3,000 rpm/min (4°C) for 15 min, and the coagulation function was determined using an automated coagulation analyzer (TECO, Germany).

#### 2.3.4. Detection of Myocardial Infarction Size

Infarct size was measured by the established method [15]. The hearts were collected, rinsed, and frozen at −20°C. The freezing hearts were cut into five specimens of ≈2 mm in thickness and incubated in 1% triphenyltetrazolium chloride (TTC) solution at 37°C for 20 minutes, followed by 4% paraformaldehyde fixation for 24 hours. The sections were photographed using a digital camera and measured with Image-Pro Plus (Media Cybernetics, Inc., Rockville, MD, USA). Infarct size is defined as the ratio of TTC-stained zones to TTC-unstained zones.

#### 2.3.5. Hematoxylin-Eosin Staining and Transmission Electron Microscopy

The heart was excised and washed in saline solution. Myocardial tissues from risk areas (approximately 2 mm in thickness) were removed. Samples were then fixed in 4% paraformaldehyde at room temperature for 1 week and embedded in paraffin for histopathological examination. Paraffin-embedded tissues were then sectioned into slices of 3 *μ*m in thickness. Sections were deparaffinized in xylene, rehydrated in graded ethanol with distilled water, and stained with hematoxylin and eosin (HE). Images were visualized under an optical microscope at ×200 magnification.

For transmission electron microscopy, myocardial tissues from risk areas (approximately 1 mm^3^) were dissected from the rats' hearts and slices were fixed in 2% glutaraldehyde for 48 h. After rinsing with the phosphate buffer, the specimens were fixed in osmium tetroxide for 2 h at room temperature. Then, the tissue specimens were dehydrated in a graded series of acetone solutions. Finally, the specimens were embedded in Araldite and sliced into ultrathin sections. The sections were imaged using a Hitachi transmission electron microscope (Hitachi, Japan) equipped for digital image acquisition.

#### 2.3.6. Real-Time Quantitative PCR

Total RNAs were isolated from myocardium using the RNeasy Mini Kit (Qiagen, #74106) and reverse transcribed into cDNA using SuperScript III Reverse Transcriptase (Thermo Fisher Scientific, #18080044). Real-time quantitative PCR for the measurements of the transcript levels of NLRP3, ASC, caspase-1, IL-1*β*, and IL-18 was performed with SYBR Green qPCR Master Mix on a real-time PCR system (Biorad, USA) and normalized to *β*-actin mRNA. The primer sequences used are in Table 2.

#### 2.3.7. Western Blot Analysis

Myocardium from the risk area (infarct margin) was used for western blot analysis. Total proteins from myocardium tissues were extracted (150 mg/lane as determined by the Bradford method) and separated by 10% sodium dodecyl sulfate-polyacrylamide gel electrophoresis (SDS-PAGE) gels. After electrophoresis, proteins were electrophoretically transferred to nitrocellulose (NC) membranes (Millipore, #Z358657), blocked with 5% BSA in Tris-buffered saline (TBS) containing 0.1% Tween 20 (TBS-T) at room temperature for 40 minutes. The membranes were then probed with primary antibodies against NLRP3, ASC, caspase-1, IL-1*β*, and *β*-actin, respectively (all 1 : 1000 diluted), at 4°C overnight. The antibody-tagged membranes were incubated with a secondary antibody solution consisting of either a 1 : 1000 dilution of horseradish peroxidase HRP-conjugated bovine antimouse IgG (for ASC) or a 1 : 1000 dilution of HRP-conjugated mouse antirabbit IgG (for NLRP3, caspase-1, IL-1*β*, and *β*-actin). An enhanced chemiluminescent (ECL) detection system was used for immunoblot proteins. The optical density of the bands (as measured in arbitrary densitometry units) was determined using Image-Pro Plus, and the densitometry of the immunoblots was normalized to *β*-actin.

### 2.4. Statistical Analysis

All results presented are representative of at least 3 independent experiments. Quantified results are expressed as mean ± SD. All data we used were normally distributed. A Student's *t*-test was used to compare the differences between two groups. One-way analysis of the variance (ANOVA) followed by Tukey's multiple comparison test was used to compare more than two groups. A value of *P* < 0.05 was considered statistically significant.

## 3. Results

### 3.1. Serum SOD and MDA Concentration

**Figure 1:**
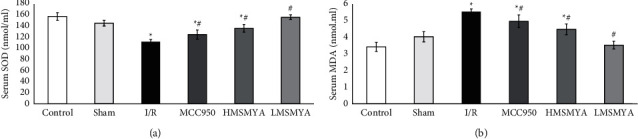
MSMYA significantly reduced myocardial oxidative stress in I/R-induced rats (*n* = 8). (a, b) Serum SOD and MDA of each group was determined using kits. Data are shown as the mean ± SD. Significance: ^*∗*^*P* < 0.05 vs. control group; ^#^*P* < 0.05 vs. I/R groups.

Serum levels of SOD and MDA are shown in Figure 1. The SOD of I/R rats showed a lower level than that of rats in the control group (*P* < 0.05). Treatment with MCC950 and different doses of MSMYA all increased SOD levels of the rats subjected to I/R (*P* < 0.05) (Figure 1(a)). The MDA of I/R rats showed a higher level than that of rats in the control group (*P* < 0.05). Treatment with MCC950 and different doses of MSMYA all decreased the MDA level of the rats subjected to I/R (*P* < 0.05) (Figure 1(b)).

### 3.2. Serum and Myocardium Tissue Homogenate Inflammatory Cytokines Caspase-1, IL-1*β*, and IL-18, and LDH Concentration

**Figure 2:**
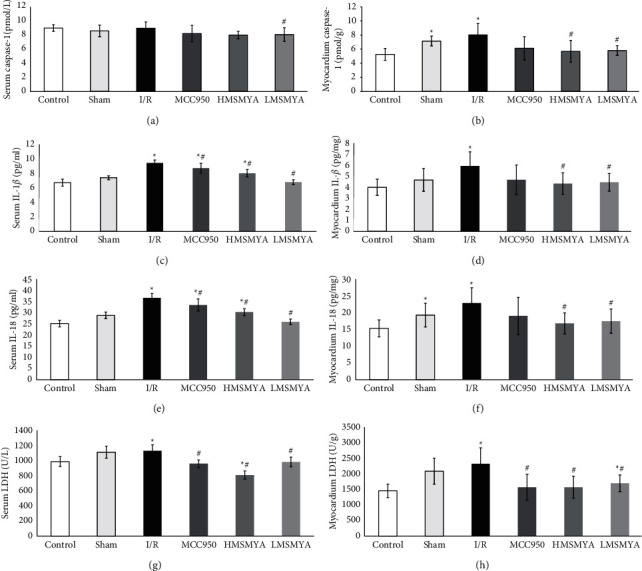
MSMYA significantly reduced myocardial injury in I/R-induced rats (n = 8). ((a), (c), (e), (g)): Serum caspase-1, IL-1β, IL-18 and LDH of each group was determined using kits; ((b), (d), (f), (h)): myocardium tissue homogenate caspase-1, IL-1β, IL-18 and LDH of each group was determined using kits. Data are shown as the mean ± SD. Significance: ^∗^P < 0.05 vs. control group; ^#^P < 0.05 vs I/R groups.

Levels of serum caspase-1, IL-1*β*, IL-18, and LDH are shown in Figures 2(a), 2(c), 2(e), and 2(g). The IL-1*β*, IL-18, and LDH levels of I/R rats all increased compared with those of rats in the control group (*P* < 0.05). Treatment with MCC950 and different doses of MSMYA all decreased caspase-1, IL-1*β*, IL-18, and LDH levels of the rats subjected to I/R (*P* < 0.05). Levels of myocardium tissue homogenate caspase-1, IL-1*β*, IL-18, and LDH are shown in Figures 2(b), 2(d), 2(f), and 2(h). The caspase-1, IL-1*β*, IL-18, and LDH levels of I/R rats all increased compared with those of rats in the control group (*P* < 0.05). Treatment with MCC950 and different doses of MSMYA all decreased caspase-1, IL-1*β*, IL-18, and LDH levels of the rats subjected to I-R (*P* < 0.05).

### 3.3. Plasma Coagulation Function

**Figure 3:**
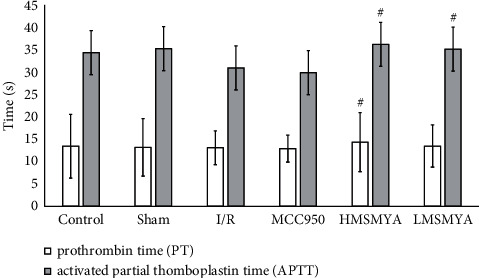
MSMYA significantly improved myocardial coagulation function in I/R-induced rats (*n* = 8). Plasma PT and APTT of each group was determined using kits. Data are shown as the mean ± SD. Significance: ^#^*P* < 0.05 vs. I/R groups.

As shown in Figure 3, compared with rats in the control group, the prothrombin time (PT) and activated partial thromboplastin time (APTT) of rats in I/R group had a decreased trend (*P* > 0.05). Compared with rats in the I/R group, PT levels of HMSMYA showed a significant increase (*P* < 0.05) and APTT levels of both HMSMYA and LMSMYA showed a significant increase (*P* < 0.05).

### 3.4. Infarct Size

**Figure 4:**
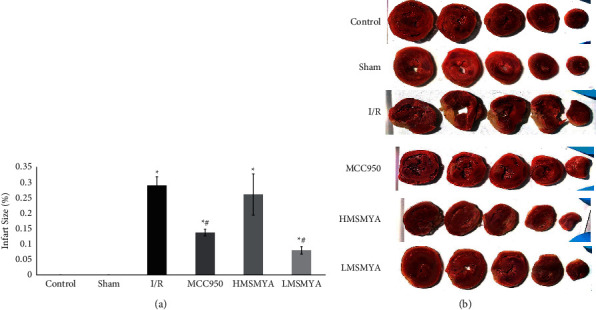
MSMYA significantly reduced myocardial infarct size in I/R-induced rats (n = 3). Data are shown as the mean ± SD. Significance: ^∗^P< 0.05 vs control group; ^#^P < 0.05 vs. I/R groups.

As shown in Figure 4, the infarct size was 29.35% in the I/R group, 13.88% in the MCC950 group, 26.38% in the HMSMYA group, and 8% in the LMSMYA group. Compared with the I/R group, treatment with MCC950 and low doses of MSMYA significantly decreased the myocardial infarct size (*P* < 0.05).

### 3.5. Myocardium Histopathological Changes

**Figure 5:**
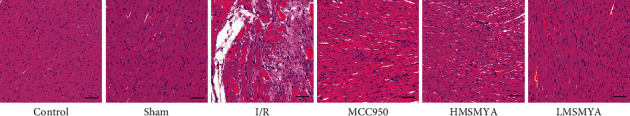
Effects of MSMYA on histopathological changes in rat heart tissues (*n* = 3 magnification ×200; scale bar 50 *μ*m).

The histopathological changes in the ischemic heart tissue were assessed by H&E staining, as shown in Figure 5. The rats in the control group showed a normal structure and shape of the myocardium. The myocardial fibers were arranged in order. The rats in the sham group featured little neutrophilic inflammation. The rats in the I/R group showed acute injury characterized by myocardial necrosis, neutrophilic inflammation, and interstitial edema. The ruptured and lysed myocardial fibers in the infarct area were found. The rats in the MCC950, HMSMYA, and LMSMYA group revealed reduced neutrophilic inflammation and interstitial edema compared with the rats in the I/R group.

### 3.6. Myocardial Ultrastructure Changes

**Figure 6:**
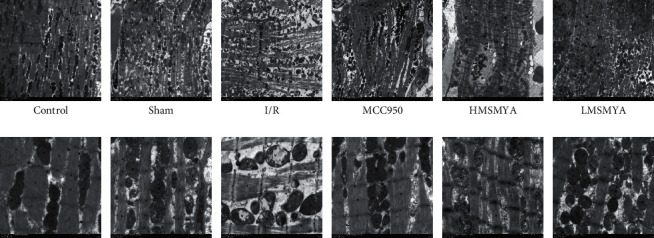
Effects of MSMYA on myocardial ultrastructure changes in rat heart tissues (*n* = 3, upper: 1200x; lower: 5000x).

As shown in Figure 6, the myocardial ultrastructure was assessed via transmission electron microscopy. The myocardial ultrastructure of control rats featured a normal ultrastructure with the characteristic linear array of sarcomeres, myofibrils, and mitochondria, an integral endoplasmic reticulum membrane, and regular nuclei. The myocardial ultrastructure of I/R rats featured severe degeneration, obvious edema in cells, decreased organelles, myofibrils mostly dissolved, broken, and large areas of focal dissolution, as well as mitochondria swelling, irregular deformation, and aggregation between myofibrils. Treatment with MCC950 and different doses of MSMYA alleviated myocardial ultrastructure injury.

The myocardial ultrastructure of sham rats and LMSMYA rats featured degeneration, moderate edema in cells, uneven thickness of myofibrils, disordered arrangement, and breakage of myofibrils, loose structure, obvious enlargement of mitochondria, breakage and reduction of mitochondrial cristae, and a small amount of aggregation between myofibrils. The myocardial ultrastructure of HMSMYA rats and MCC950 rats featured mild edema, abundant organelles and disordered arrangement of myofibrils, and slightly swollen mitochondria.

### 3.7. Relative mRNA Expression of NLRP3 Inflammasome Components

**Figure 7:**
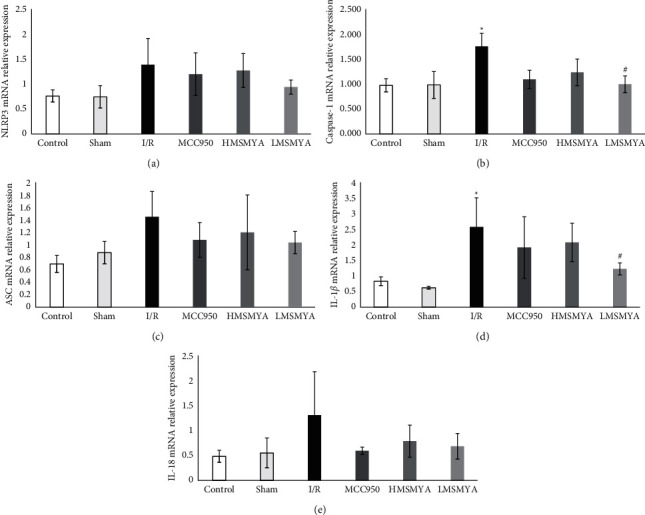
Effects of MSMYA on NLRP3 inflammasome-related molecular mRNA relative expression in I/R-reduced rats (*n* = 3). (a) NLRP3 mRNA relative expression; (b) caspase-1 mRNA relative expression; (c) ASC mRNA relative expression; (d) IL-1*β* mRNA relative expression; (e) IL-18 mRNA relative expression. Data are shown as the mean ± SD. Significance: ^*∗*^*P* < 0.05 vs. control group; ^#^*P* < 0.05 vs. I/R groups.

As shown in Figure 7, to determine the effects of MSMYA on the activation of NLRP3 inflammasome in the I/R rat model, we examined the mRNA relative expression of the inflammasome components, including NLRP3, ASC, caspase-1, IL-1*β,* and IL-18. The NLRP3, caspase-1, ASC, IL-1*β*, and IL-18 levels of I/R rats all increased compared with those of rats in the control group, while caspase-1 and IL-1*β* had a significantly difference (*P* < 0.05). Treatment with MCC950 and different doses of MSMYA all decreased NLRP3, caspase-1, ASC, IL-1*β*, and IL-18 mRNA expressions compared with rats subjected to I/R , while only caspase-1 and IL-1*β* mRNA expressions in the LMSMYA group showed a significant decrease compared with those in the I/R group (*P* < 0.05).

### 3.8. NLRP3, ASC, and IL-1*β* Protein Expression as Well as Caspase-1 Activation

**Figure 8:**
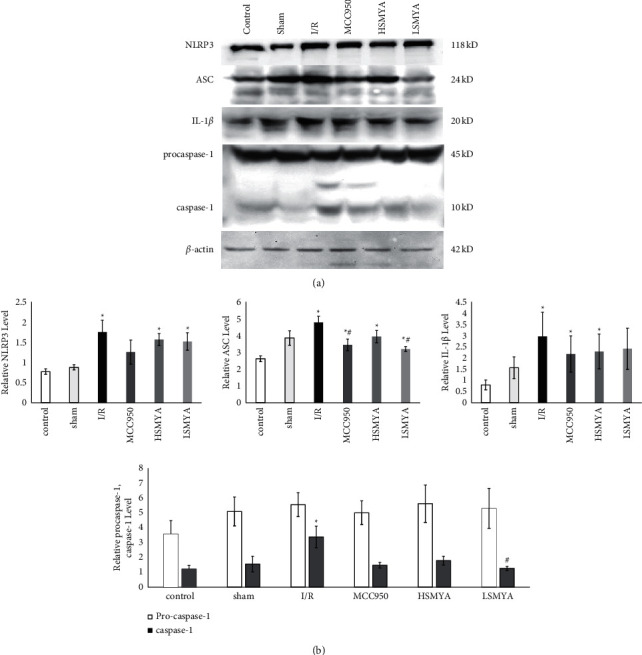
Effects of MSMYA on NLRP3 inflammation pathway in I/R-reduced rats. ((a), (b)): Protein levels of NLRP3, ASC, IL-1β, caspase-1 in each group by western blot. Data are shown as the mean ± SD. Significance: ^∗^P < 0.05 vs control group; ^#^P < 0.05 vs. I/R groups.

As shown in Figure 8, compared with the control group, I/R increased NLRP3, ASC, IL-1*β,* and activated caspase-1 protein expressions (*P* < 0.05), while NLRP3 and IL-1*β* protein expressions showed a decreasing tread in treatment groups compared with the I/R group (*P* > 0.05). ASC protein expression was significantly reduced in MCC950 and low doses of the MSMYA group (*P* < 0.05). Activated caspase-1 protein expression was significantly reduced in low doses of the MSMYA group (*P* < 0.05). Protein expression of procaspase-1 was not significantly different among the six groups (*P* > 0.05).

## 4. Discussion

Ischemia/reperfusion (I/R) injury refers to the progressive and irreversible injury caused by reperfusion after a certain period of ischemia, which is still the main reason resulting in long-term mortality and the poor quality of life in coronary heart disease (CHD) patients [16]. Chinese medicine has been proved an effective treatment for I/R injury.

In Chinese medicine, I/R injury is usually called “chest pain or palpitation.” The root pathogenesis of I/R is deficiency in origin (qi, blood, yin, and yang) and excess in superficiality (phlegm, blood stasis, heat, and toxin). The treatment principle should be based on activating blood circulation to remove blood stasis, clearing heat and detoxification, and supplementing qi and nourishing yin. Recent studies have shown that inflammation is a key pathological mechanism of myocardial I/R injury and closely related to the development and prognosis of this disease. Inflammation inhibition can be used as a new treatment to improve cardiac injury.

Si-Miao-Yong-An decoction is a classic prescription of gangrene. Moreover, its cardiovascular protective effects have been proved by modern pharmacological research studies [17]. Our research study used modified Si-Miao-Yong-An (MSMYA), which is based on the original prescription but adding supplementing qi and nourishing yin herb “American ginseng.” The fundamental pathogenesis of myocardial ischemia/reperfusion is “deficiency in origin and excess in superficiality.” Therefore, supplement (qi or yin) deficiency should be strengthened as well as heat and detoxification should be cleared. As the “The Yellow Emperor's Canon of Internal Medicine” mentioned that “as long as the healthy Qi is stored inside, the pathogenic factors cannot invade.”

Inflammatory cascade reaction is an important mechanism of reperfusion injury aggravation, which plays an important role in the process of myocardial I/R [18]. As the core of the inflammatory reaction, NLRP3 inflammasome has been deeply explored [19]. A large number of experimental studies have shown that inhibiting the activation of NLRP3 inflammasome has protective effects against myocardial I/R injury. Clinical studies have also confirmed that NLRP3 overexpressed in the aorta of patients with coronary heart disease patients undergoing coronary artery bypass grafting, and NLRP3 expression was positively correlated with the severity of coronary heart disease [20–22].

In our study, we used the I/R rat model and intervened with MSMYA to investigate the decoction myocardial protective effects and potential mechanisms from aspects of oxidative stress, inflammatory reaction, coagulation function, morphology, and NLRP3 inflammasome signaling pathways.

Oxidative stress can be reflected by testing superoxide dismutase (SOD) and malondialdehyde (MDA) [23]. Our study showed that the serum SOD level in the model group was significantly decreased, and the serum MDA level was significantly increased. NLRP3 inhibitor and MSMYA could improve oxidative stress reaction by increasing SOD and decreasing MDA. Compared with the NLRP3 inhibitor group, the improvement of the serum oxidative stress factor in different doses of the MSMYA group was more obvious.

During myocardial reperfusion injury, a large number of reactive oxygen species (ROS) were produced and accumulated, which induced the activation of NLRP3 inflammasome. The pro-IL-1*β* and pro-IL-18 were continuously cleaved to mature IL-1*β* and IL-18, respectively, and as the integrity of the cell membrane was destroyed, the contents of the cell were dissolved and released. IL-18 and IL-1*β* were further released to recruit more inflammatory cells, expand the inflammatory response, and release a large number of inflammatory substances, leading to the occurrence of inflammatory cascade, cell edema, death and the generation of free radicals, and aggravating myocardial injury [24]. Our study showed that MSMYA intervention can alleviate inflammatory cytokine release.

Furthermore, coagulation dysfunction is an important indicator of I/R injury. The four coagulation reactions are commonly applied to assay coagulation function. The decrease in PT and APTT could reflect the hypercoagulability of blood [25]. Our results showed I/R led to coagulation dysfunction, which indicated a hypercoagulable state. While those indicators were increased among the normal value range after MSMYA treatment, which indicated that MSMYA can improve coagulation function through regulating the activation of coagulation factors.

Myocardial I/R can directly cause myocardial damage, which can be reflected by chemical injury, factor release, infarct size, histopathological, and ultrastructural changes. Inflammatory factors aggravated cell membrane damage, leading to LDH leakage, which further aggravated damage. As shown in our study, the I/R group aggravated myocardial damage by increasing LDH leakage, infarct size, and exacerbating myocardial ultrastructural damage. Furthermore, NLRP3 inhibitors and different doses of MSMYA can decrease LDH levels and infarct size in varying degrees, reduce intracellular edema, mitochondrial swelling, and cristae rupture, as well as improving myofibrillar arrangement, indicating that MSMYS can relieve myocardial ischemia/reperfusion injury by improving cardiac structure.

In addition, we further detected the mRNA and protein expression of NLRP3 pathway-related molecules to explore relevant protective mechanisms and found that reperfusion injury-activated NLRP3 inflammatory pathway. However, NLRP3 inhibitors and different doses of MSMYA could reduce caspase-1 and IL-1*β* mRNA expression, and especially the low-dose group. Meanwhile, we found that MSMYA has shown a significant cardioprotective effect against I/R injury through inhibiting NLRP3 inflammatory pathway as shown by the reduction in NLRP3, ASC, and IL-1*β* protein expressions, as well as caspase-1 activation, which is closely associated with inflammatory cytokine release. According to our research, the protective effect of MSMYA is not dose-dependent completely. To evaluate myocardial injury aspects, which are including oxidative stress, inflammatory cytokines and LDH release, coagulation function, and infarct size, a low dose is better than a high dose, indicating that LMSMYA is unified with clinic proper dosage application. While in histopathological and ultrastructural changes, a high dose is better than a low dose, indicating that HMSMYA probably has underlying advantages in further improving cardiomyocytes necrosis or apoptosis. We will continue to investigate the specific mechanism in our further research.

## 5. Conclusion

In conclusion, our results demonstrated that the NLRP3 inflammasome played a vital role in response to reperfusion injury, and we initially identified novel mechanisms by which MSMYA inhibits NLRP3 inflammasome activation. Our findings may aid the development of TCMs in treating cardiovascular diseases.

## Figures and Tables

**Table 1:** Information of components in modified Si-Miao Yong-An decoction (MSMYA).

Botanical name	Herbal name	Chinese name	Medical parts	Ratio
*Lonicera japonica* Thunb.	Lonicerae japonicae flos	Jin-Yin-Hua	Flower	5
*Scrophularia ningpoensis* Hemsl.	Scrophulariae Radix	Xuan-Shen	Root	4
*Angelica sinensis* (Oliv.) Diels	Angelicae sinensis radix	Dang-Gui	Root	4
*Glycyrrhiza* uralensis Fisch.	Glycyrrhizae Radix et Rhizoma	Gan-Cao	Root	3
*Panax quinquefolius* L.	Radix Panacis Quinquefolii	Xi-Yang-Shen	Root	5

**Table 2:** Real-time PCR primer sequence.

Gene	Forward sequence (5′-3′)	Length	nmol/OD
R-caspase-1-F	TGCCTGGTCTTGTGACTTGGAG	132	4.45
R-caspase-1-R	TGTCCTGGGAAGAGGTAGAAACG		3.7
R-GAPDH-F	CTGGAGAAACCTGCCAAGTATG	138	4
R-GAPDH-R	GGTGGAAGAATGGGAGTTGCT		4.07
R-NLRP3-F	TCTTTGCGGCTATGTACTATCT	113	4.59
R-NLRP3-R	TTCTAATAGGACCTTCACGT		4.75
R-IL-18-F	GGAATCAGACCACTTTGGCAGA	149	4
R-IL-18-R	GTCTGGTCTGGGATTCGTTGG		4.69
R-IL-1*β*-F	ACTTGGGCTGTCCAGATGAGAG	203	4.1
R-IL-1*β*-R	CGAGTCACAGAGGACGGGCT		4.45
R-ASC-F	CTGTGCTTAGAGACATGGGCA	95	4.88
R-ASC-R	AGGGACACTGGTTGCAGTAG		4.96

## Data Availability

The data used to support the findings of this study are included in the article.

## Supplementary Materials

Supplementary Materials The original western blots (in triplicate) data for analysis are shown in the supplementary files.
