# Supplementary figures and images for: Therapeutic Effects of Modified Si-Miao-Yong-An Decoction in the Treatment of Rat Myocardial Ischemia/Reperfusion Injury
Source: Evid Based Complement Alternat Med. 2022 Jun 6;2022:1442405. doi: 10.1155/2022/1442405 (PMC9192308; doi:10.1155/2022/1442405)

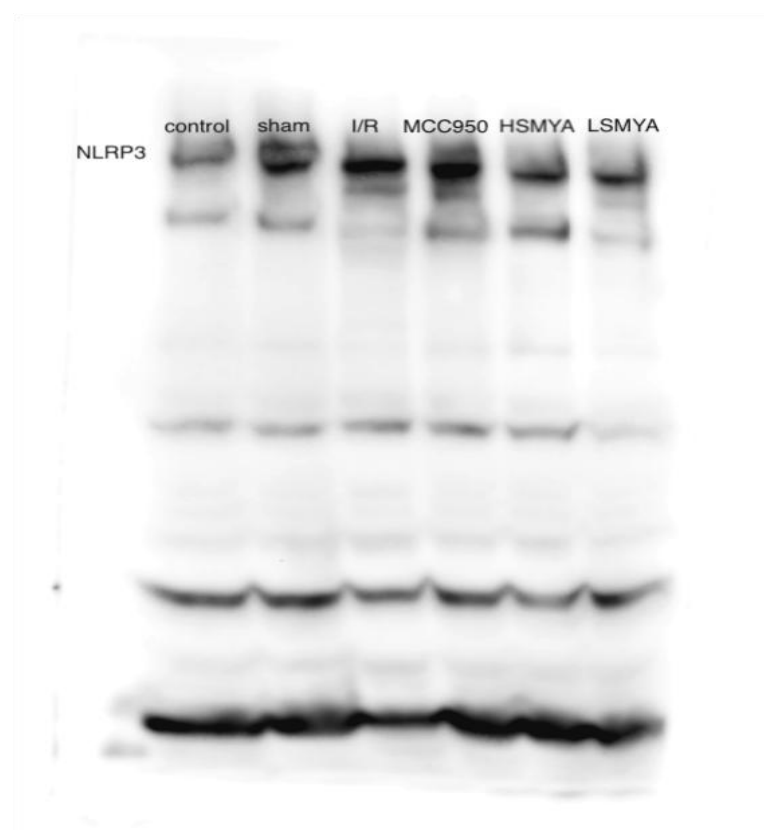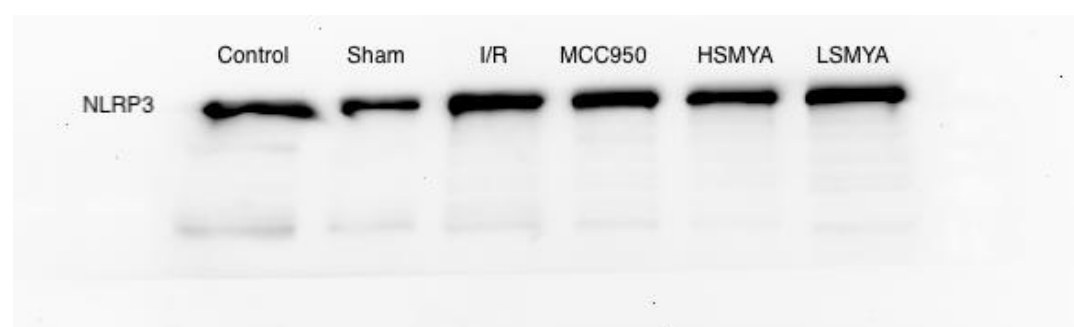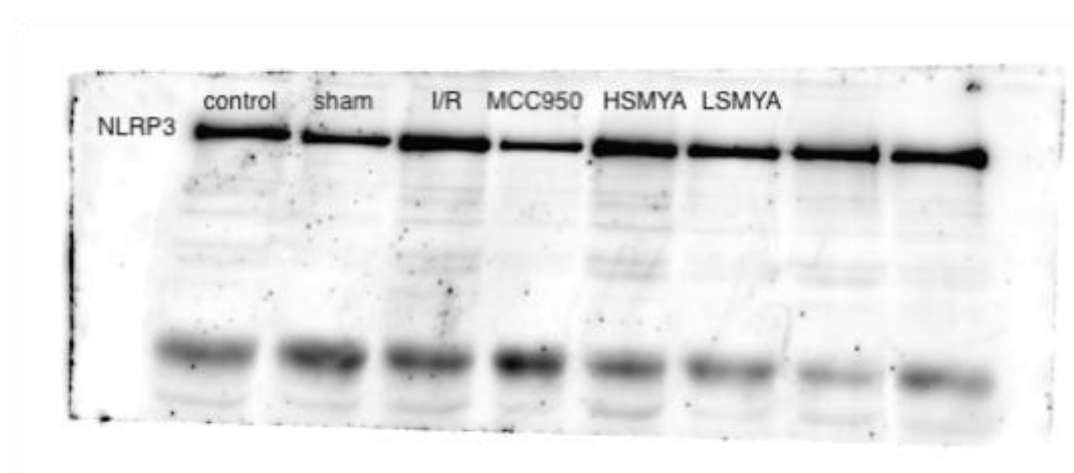

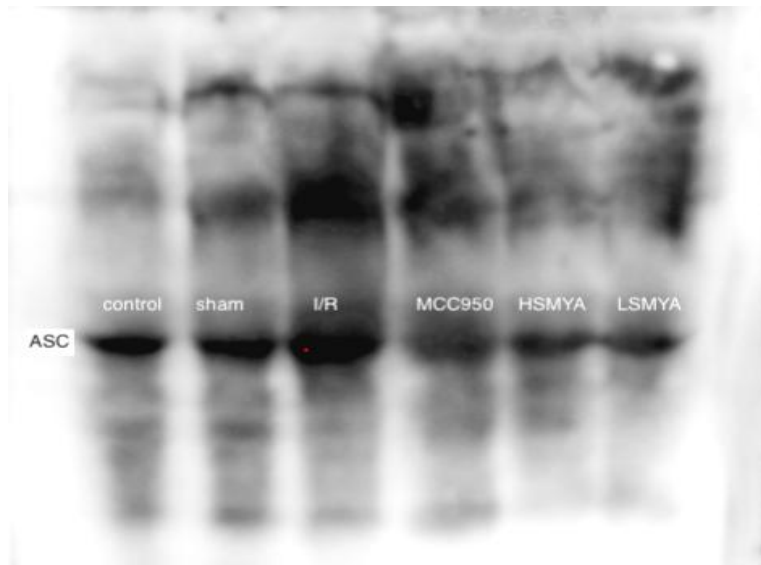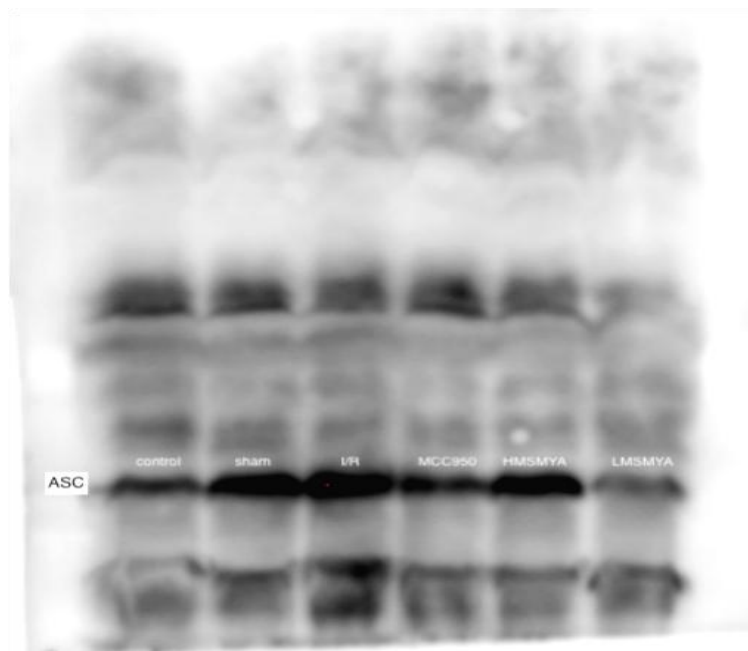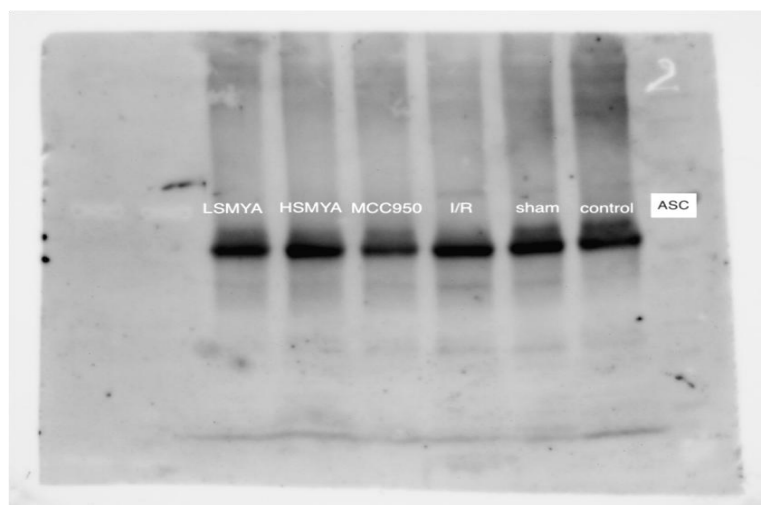

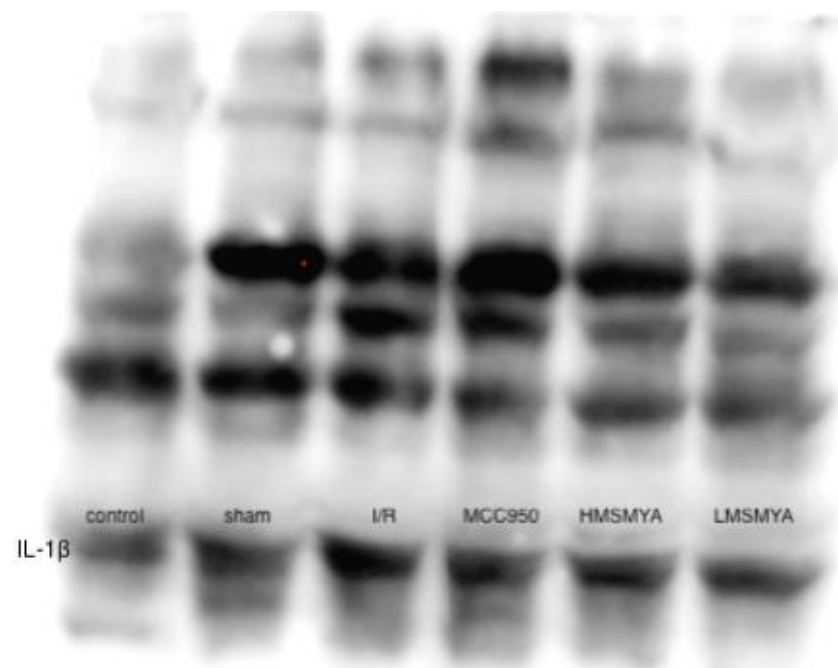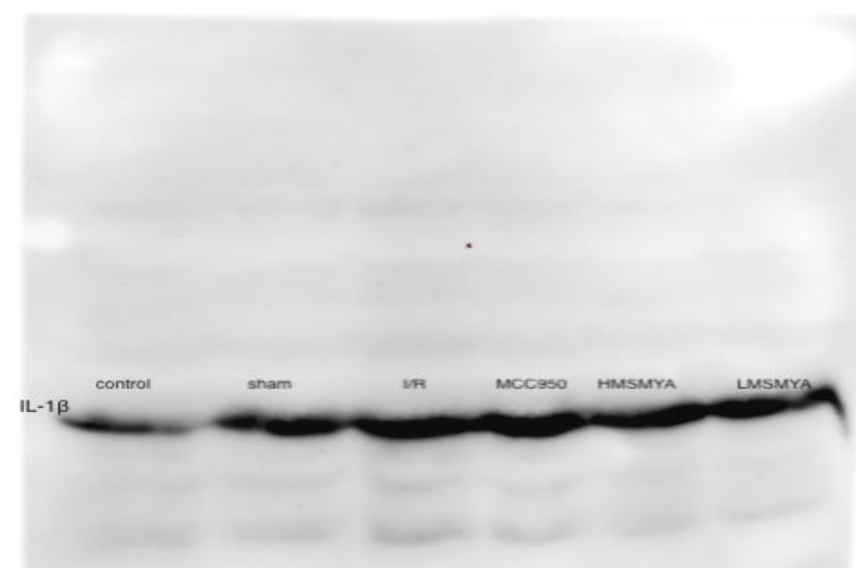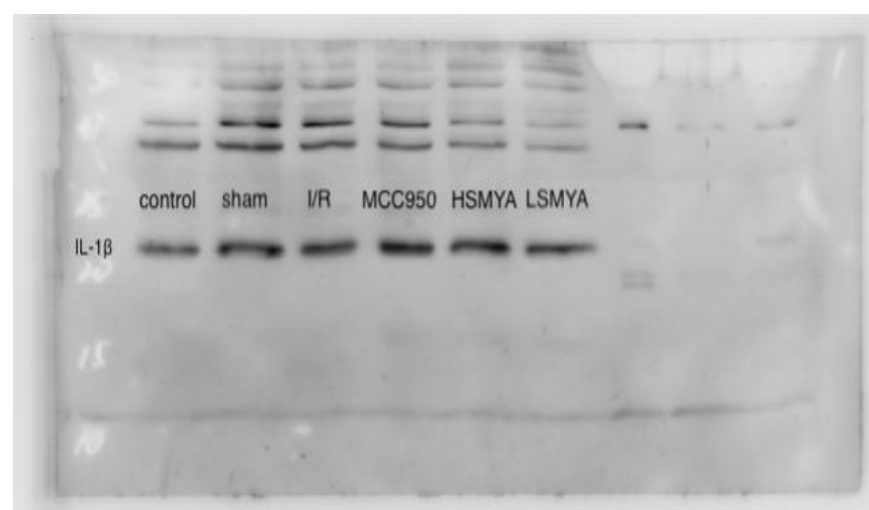

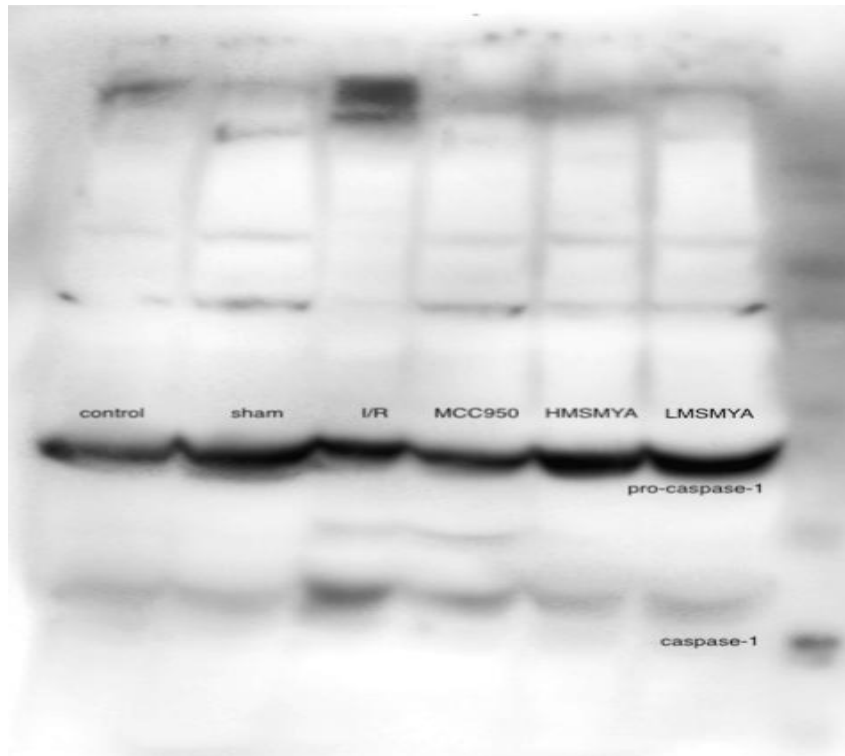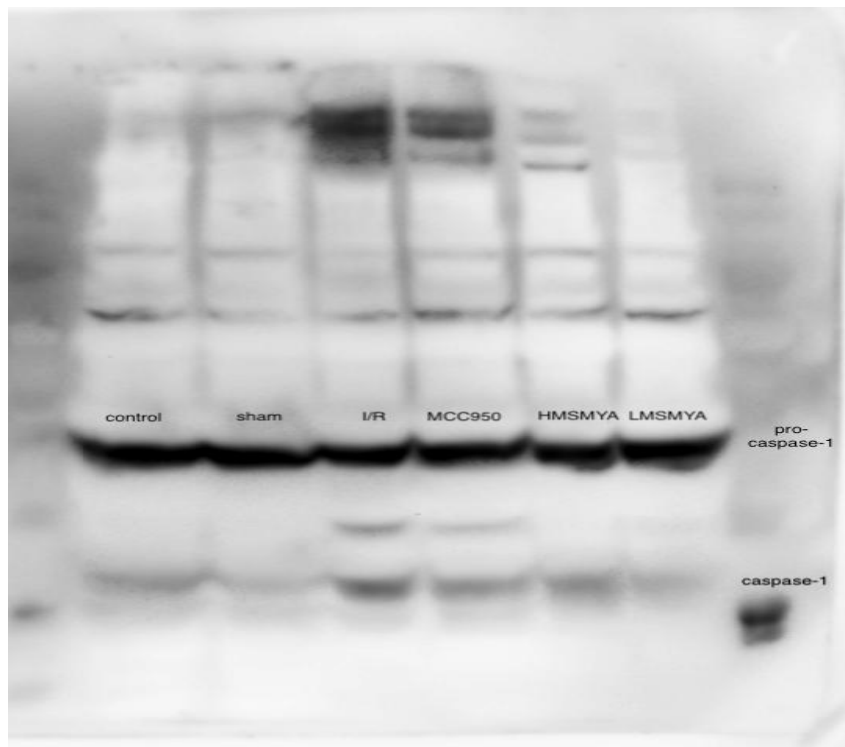

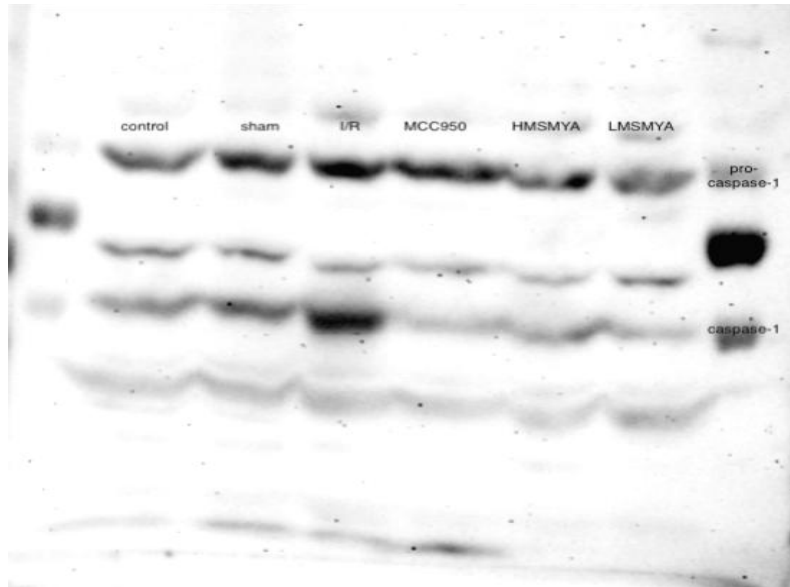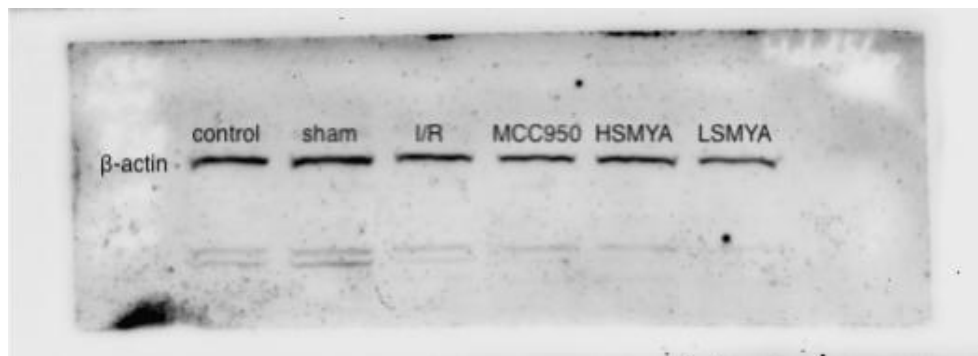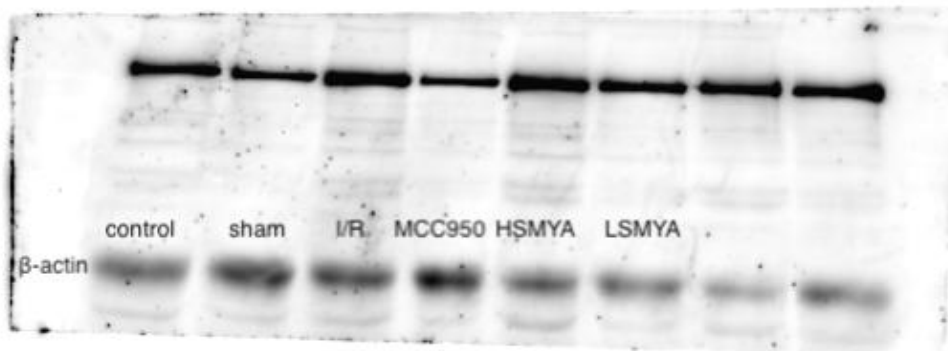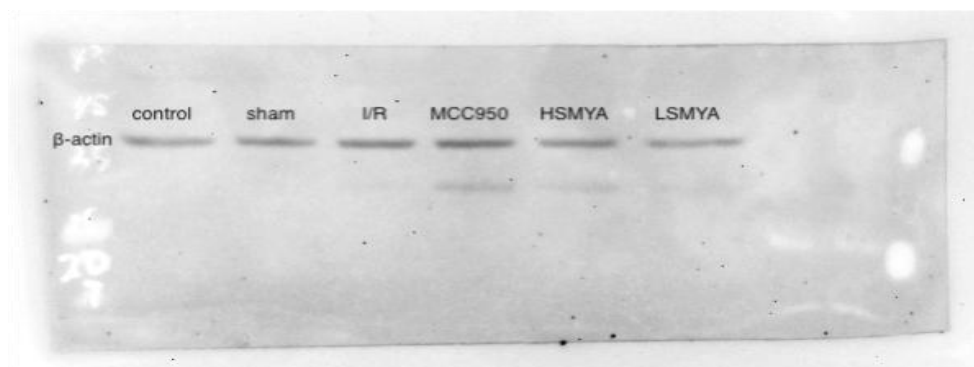

Supplement: Supplementary Materials — The original western blots (in triplicate) data for analysis are shown in the supplementary files. [file 1442405.f1.pdf]
